# The efficacy and safety of Xuebijing injection in the treatment of radiation pneumonitis: A protocol for systematic review and meta-analysis

**DOI:** 10.1097/MD.0000000000024344

**Published:** 2021-02-05

**Authors:** Zheng Li, Dandan Wang, Ying Zhang, Shuo Wang, Xueqian Wang, Yuxiao Li, Yuerong Gui, Jun Dong, Wei Hou

**Affiliations:** Guang’an Men Hospital, China Academy of Chinese Medical Sciences, Beijing, China.

**Keywords:** efficacy and safety, meta-analysis, radiation pneumonitis, randomized controlled trials, Xuebijing injection

## Abstract

**Background:**

At present, the treatment of radiation pneumonitis (RP) is still a clinical problem. Although a variety of drugs such as glucocorticoids and antibiotics are used for RP treatment, side effects remain to be inevitable. Xuebijing injection (XBJ), a Chinese herbal injection, has been widely used in RP treatment, but there is no published systematic review to evaluate its efficacy and safety.

**Methods:**

Based on Preferred Reported Items for Systematic Review and Meta-analysis guidelines, we will conduct this systematic review and meta-analysis. Related randomized controlled trials will be searched in 7 databases: PubMed, Cochrane Library, EMBASE, China National Knowledge Infrastructure, WANFANG database, SinoMED, and China Science and Technology Journal Database from inception of the library to October 1, 2020. Two researchers will independently carry out literature screening, data extraction, and bias risk assessment. The primary outcome is total effective rate and the secondary outcome is inflammation-related indicators such as C-reactive protein, tumor necrosis factor alpha, interleukin-6, interleukin-10, etc and adverse events. Cochrane Review Manager (RevMan 5.3) software will be applied to analyze the data and generate forest plot and funnel plot.

**Results:**

This study will provide a synthesis of current evidence of XBJ in RP treatment including total effective rate and inflammation-related indicators such as CRP, TNF-α, IL-6, IL-10, etc. and adverse events (AEs).

**Conclusion:**

This systematic review and meta-analysis will objectively evaluate the efficacy and safety of XBJ in the treatment of RP, and provide evidence for the application of XBJ in RP treatment.

**Systematic review registration number:**

INPLASY2020120037

## 1 Introduction

Radiotherapy is one of the main methods to treat thoracic neoplasms. When receiving chest radiotherapy, it is easy to develop radiation pneumonitis (RP) because the lung tissue is moderately sensitive to radiotherapy. The incidence of RP varies from about 5% to 50% due to the different definition of clinically significant lung injury^[1]^ and different kinds of cancer.^[2–4]^ With the application of intensity modulated radiation therapy, the incidence is still as high as 30%.^[5,6]^ In addition, RP may cause radiation-induced fibrosis or lung failure, life-threatening symptoms, and situations.^[7]^ Therefore, reducing the risk of RP is essential to improve tumor control and quality of life of the patients.^[8]^

The current treatment of RP is mainly based on glucocorticoids,^[9]^ along with bronchodilators, antibiotics, and respiratory support treatment.^[10]^ However, long-term use of glucocorticoids can cause side effects such as immunosuppression, central obesity, osteoporosis, and peptic ulcers, which affect quality of life of the patients. And if RP develops to radiation-induced fibrosis, the effectiveness of glucocorticoids is greatly reduced.^[11]^ In this case, finding effective therapeutic drugs with low side effects and complementary and alternative drugs that can reduce the side effects of glucocorticoids will be of great significance to the patients.

Xuebijing injection (XBJ), a Chinese herbal injection, has been widely used for the treatment of RP in clinical practice. It is composed of *Carthamustinctorius* flowers, *Paeonialactiflora* roots, *Ligusticum* chuanxiong rhizomes, *Angelica sinensis* roots and *Salvia miltiorrhiza* roots.^[12]^ XBJ has been regarded as potential anti-inflammatory and immune regulation effects.^[13–14]^ Modern pharmacology has proved that its active ingredients include safflower yellow A, ligustrazine, danshensu, ferulic acid, paeoniflorin, protocatechualdehyde, etc,^[15]^ which can antagonize endotoxin and reduce rat mortality. At present, there are many clinical studies of XBJ, but its effectiveness and safety for RP have not been objectively evaluated. Therefore, this article aims to systematically evaluate the efficacy and safety of XBJ in the treatment of RP and provide objective evidence for clinical use.

## 2 Methods

### 2.1 Study registration

This protocol has been registered on INPLASY (ID:INPLASY2020120037, (https://inplasy.com/inplasy-2020-12-0037/) and will be conducted based on the Preferred Reporting Items for Systematic Reviews and Meta-Analysis Protocol statement guidelines.^[16]^ Because all the research materials are published studies, this study does not require ethical approval.

### 2.2 Inclusion and exclusion criteria for this review

#### 2.2.1 Types of studies

Only randomized controlled trials (RCTs) will be eligible for inclusion regardless of the languages. It will not include animal experiments, case reports, non-clinical researches, commentaries, repeated publications, etc RCTs with incomplete and unavailable important data will be excluded.

#### 2.2.2 Type of participants

The study will recruit participants over 18 years of age who met the histological or pathological or clinical diagnostic criteria for RP.^[17]^ There will be no restrictions on gender, nationality, race, education, and job.

#### 2.2.3 Type of interventions

The interventions will contain 2 groups: experimental group and control group. The interventions of control group will include glucocorticoids, bronchodilators, antibiotics, oxygen therapy, and respiratory support treatment if necessary. The interventions of experimental group will include the interventions of control group and XBJ.

#### 2.2.4 Primary outcomes

The primary outcome is the total effective rate, which contains the cure rate and the remission rate. The calculation method is equal to the ratio of the number of patients cured and relieved to the total number of patients.^[18]^

#### 2.2.5 Secondary outcomes

The secondary outcome will consist of inflammation-related indicators such as C-reactive protein, tumor necrosis factor alpha, interleukin-6, interleukin-10, etc and adverse events.

### 2.3 Search methods and strategy

We will comprehensively search 7 databases: PubMed, Cochrane Library, EMBASE, China National Knowledge Infrastructure, WANFANG database, SinoMED, and China Science and Technology Journal Database. There is no limitation of the language. The time interval for literature searching will be from inception of the library to October 1, 2020. The search items will be composed of the following key words: Radiation Pneumonitis, Xuebijing injection, and Randomized Controlled Trial. Table 1 showed the details of search strategy for PubMed as an example.

**Table 1:** Search strategy for PubMed.

Number	Search terms
#1	Radiation pneumonitis[MeSH]
#2	(radiation pneumonitides) or (pneumonitides, radiation) or (pneumonia, radiation) or (pneumonias, radiation) or (radiation pneumonias) or (pneumonitis, radiation) or (radiation pneumonia) or (fibrosis, radiation) or (radiation fibrosis) or (radiation induced lung injury) or (radiation induced pneumonitis)
#3	(Xuebijing) or (Xue-Bi-Jing) or (Xuebijing injection) Or (Xue-Bi-Jing injection)
#4	(randomized controlled trials[MeSH])or (clinical trials, randomized) or (trials, randomized clinical) or (controlled clinical trials, randomized) or (RCT)
#5	#1 and #2 and #3 and #4

### 2.4 Literature screening and selection

**Figure 1:**
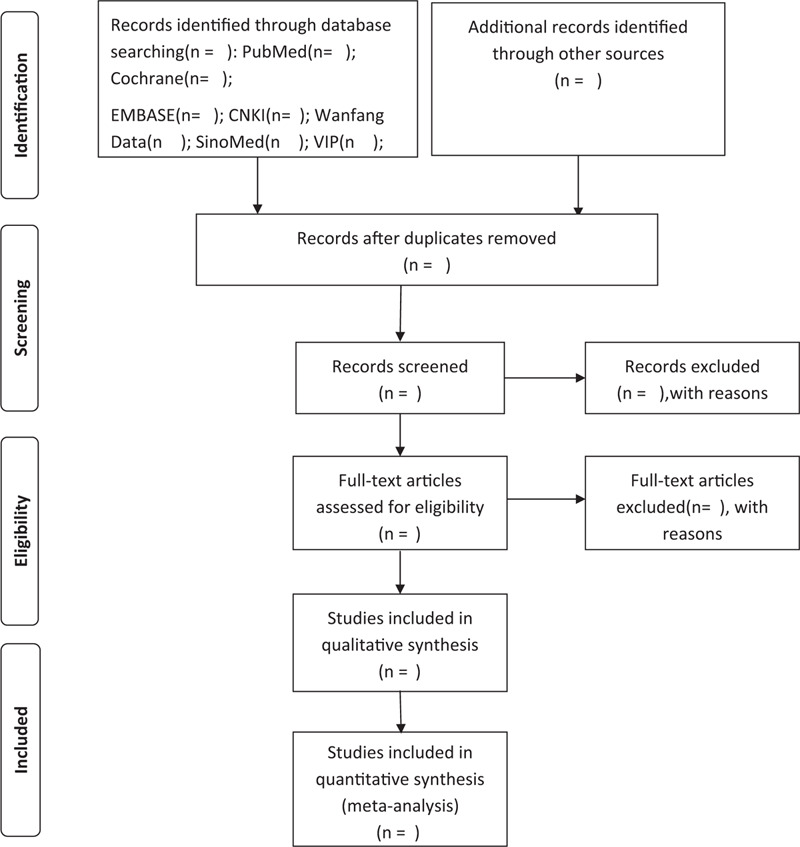
Flow chart of study selection process.

The literature screening and selection process will be conducted independently by 2 reviewers. The titles of the literature will be screened first and the duplicates will be deleted. According to the inclusion and exclusion criteria, the abstracts and articles will be read carefully and then the articles that do not meet the criteria will be eliminated. If there are disagreements in the process, a third reviewer will assist in the evaluation. NOTEEXPRESS (version 3.0, http://www.inoteexpress.com/aegean/index.php/home/index/index.html, AEGEAN Co., Ltd., Beijing, China) will be applied to manage literature. The details of literature screening and selection process were shown in Figure 1.

### 2.5 Data processing and analysis

#### 2.5.1 Data extraction

Two reviewers will independently extract data from the selected articles and fill in the data sheet. If there is any objection, it will be resolved through discussion or negotiation with the third reviewer. The extracted items are as follows: year of publication, first author, country, research design, sample size, age, gender, intervention measures, dosage and usage, primary and secondary outcomes, adverse events.

#### 2.5.2 Dealing with missing data

If there is incomplete information, we will try our best to contact the original author to obtain the missing data. If the original author cannot be contacted, the study will be eliminated.

#### 2.5.3 Risk of bias assessment

Two reviewers will use the “risk of bias assessment tool” of the Cochrane Handbook to evaluate the risk of bias of the included RCTs.^[19]^ The evaluation criteria include the method of random sequence generation, allocation concealment, blinding of patients, personnel and assessors, incomplete outcome data, selective reporting, and other sources of bias. Any disagreement will be resolved by the third reviewer.

#### 2.5.4 Data synthesis and analysis

Data synthesis and analysis will be conducted by Revman software (version 5.3, Copenhagen: The Nordic Cochrane Center, The Cochrane Collaboration). Different data types will be processed in different ways: continuous data and dichotomous data will be evaluated by standard mean difference with 95% confidence interval and rate ratio with 95% confidence interval respectively. The heterogeneity will be judged by the I^2^ value. For I^2^<50% and I^2^>50%, the fixed effects model and the random effects model will be used respectively. If the quantitative synthesis of data is not possible, qualitative analysis will be applied.

#### 2.5.5 Subgroup and sensitivity analysis

If I^2^>50%, we will also explore the source of heterogeneity by subgroup and sensitivity analysis. We will conduct subgroup analysis based on interventions, participants characteristics, and outcome measurement if the included articles are at least 10. In the sensitivity analysis, we will remove each study in turn to observe the impact on the overall results.

#### 2.5.6 Publication bias assessment

If there are more than 10 articles for meta-analysis, we will generate a funnel plot to assess publication bias.^[20]^

## 3 Discussion

Currently, RP is still based on anti-inflammatory treatments such as glucocorticoids. As complementary and alternative medicine, TCM plays a potential role in the treatment of RP. In China, XBJ combined with glucocorticoids can not only improve the efficacy and relieve symptoms, but also reduce the side effects of glucocorticoids. However, as far as we know, XBJ is mainly used in TCM hospitals in China, and there is no systematic review to evaluate its efficacy and safety. Therefore, we will implement this systematic review to provide best evidence for clinical decision of using XBJ and to provide complementary alternative therapy for the treatment of RP.

Although the design of systematic review follows Preferred Reported Items for Systematic Review and Meta-analysis guideline, there may still be potential deficiencies. First of all, XBJ has been widely used in China, which may lead to regional bias. Then, the number of literature to be searched may be small, resulting in some indicators that cannot be combined and analyzed. Thirdly, although the components of XBJ are relatively clear, the mechanism of relieving RP is still unknown, thus hindering its popularization.

## Author contributions

**Conceptualization:** Xueqian Wang, Ying Zhang, Wei Hou.

**Data curation:** Zheng Li, Dandan Wang.

**Methodology:** Shuo Wang, Yuxiao Li.

**Resources:** Yuerong Gui, Jun Dong.

**Supervision:** Ying Zhang, Wei Hou.

**Writing – original draft:** Zheng Li, Dandan Wang.

**Writing – review & editing:** Ying Zhang.
